# Standardization of the [^68^Ga]Ga-PSMA-11 Radiolabeling Protocol in an Automatic Synthesis Module: Assessments for PET Imaging of Prostate Cancer

**DOI:** 10.3390/ph14050385

**Published:** 2021-04-21

**Authors:** Leonardo L. Fuscaldi, Danielle V. Sobral, Ana Claudia R. Durante, Fernanda F. Mendonça, Ana Cláudia C. Miranda, Marcelo L. da Cunha, Luciana Malavolta, Jorge Mejia, Marycel F. de Barboza

**Affiliations:** 1 Hospital Israelita Albert Einstein, Sao Paulo 05652-900, Brazil; leonardo.fuscaldi@hotmail.com (L.L.F.); aninhapharma30@gmail.com (A.C.R.D.); ana.miranda@einstein.br (A.C.C.M.); marcelo.cunha@einstein.br (M.L.d.C.); jorge.mecabeza@einstein.br (J.M.); 2 Department of Physiological Sciences, School of Medical Sciences, Santa Casa de Sao Paulo, Sao Paulo 01221-020, Brazil; danielle_sobral@hotmail.com (D.V.S.); fernandaferreiramendonca@hotmail.com (F.F.M.); luciana.malavolta@gmail.com (L.M.)

**Keywords:** PSMA-11, gallium-68, automatic synthesis module, PET imaging, prostate cancer

## Abstract

Prostate-specific membrane antigen (PSMA) is a glycoprotein present in the prostate, that is overexpressed in prostate cancer (PCa). Recently, PSMA-directed radiopharmaceuticals have been developed, allowing the pinpointing of tumors with the Positron Emission Tomography (PET) or Single Photon Emission Computed Tomography (SPECT) imaging techniques. The aim of the present work was to standardize and validate an automatic synthesis module-based radiolabeling protocol for [^68^Ga]Ga-PSMA-11, as well as to produce a radiopharmaceutical for PET imaging of PCa malignancies. [^68^Ga]Ga-PSMA-11 was evaluated to determine the radiochemical purity (RCP), stability in saline solution and serum, lipophilicity, affinity to serum proteins, binding and internalization to lymph node carcinoma of the prostate (LNCaP) cells, and ex vivo biodistribution in mice. The radiopharmaceutical was produced with an RCP of 99.06 ± 0.10%, which was assessed with reversed-phase high-performance liquid chromatography (RP-HPLC). The product was stable in saline solution for up to 4 h (RCP > 98%) and in serum for up to 1 h (RCP > 95%). The lipophilicity was determined as −3.80 ± 0.15, while the serum protein binding (SPB) was <17%. The percentages of binding to LNCaP cells were 4.07 ± 0.51% (30 min) and 4.56 ± 0.46% (60 min), while 19.22 ± 2.73% (30 min) and 16.85 ± 1.34% (60 min) of bound material was internalized. High accumulation of [^68^Ga]Ga-PSMA-11 was observed in the kidneys, spleen, and tumor, with a tumor-to-contralateral-muscle ratio of >8.5 and a tumor-to-blood ratio of >3.5. In conclusion, an automatic synthesis module-based radiolabeling protocol for [^68^Ga]Ga-PSMA-11 was standardized and the product was evaluated, thus verifying its characteristics for PET imaging of PCa tumors in a clinical environment.

## 1. Introduction

Prostate cancer (PCa) is the second most prevalent type of cancer among men, with an incidence rate of 109.8 new cases/100,000 individuals each year from 2013 to 2017 in the US, and with 191,930 new cases estimated for 2020 [1]. In Brazil, according to data from the National Cancer Institute for 2020, 65,840 new cases of PCa are estimated, which corresponds to a gross rate of 66.12 cases/100,000 individuals [2].

The prostate-specific membrane antigen (PSMA) is a type-II transmembrane glycoprotein that has several enzymatic functions. This antigen is expressed in the prostatic tissue, as well as in other organs, such as the small intestine, kidneys, liver, brain, salivary glands, and lacrimal glands [3]. However, PSMA is overexpressed in the PCa tissue in direct association with the cancer’s aggressiveness, in lymph nodes and bone metastases, and also in other castration-resistant PCa lesions [4,5,6,7], with levels reaching from 100 to 1000 times the values found in non-cancerous prostatic cells [8]. It was previously detected in the lymph node carcinoma of the prostate (LNCaP) human cell line [9]. Given all of these factors, PSMA is considered an appropriate biomarker for PCa and an attractive target for theranostic applications [10].

Since 2001, small-sized PSMA-inhibiting molecules that interact with the binuclear active site of zinc have been developed, successfully radiolabeled, and tested to obtain Single Photon Emission Computed Tomography (SPECT) and Positron Emission Tomography (PET) images of PCa tumors [11]. These small molecules have been labeled with different radioisotopes (^99m^Tc, ^111^In, ^18^F, or ^68^Ga) to produce, for example, [^99m^Tc]Tc-MIP-1404, [^99m^Tc]Tc-PSMA-I&S, [^111^In]In-PSMA-I&T, [^18^F]F-FDCFPyL, [^18^F]F-PSMA-1007, or [^68^Ga]Ga-PSMA-HBED-CC. Almost simultaneously, the labeling of those molecules with particle-emitting radioisotopes, such as ^177^Lu ([^177^Lu]Lu-PSMA-617, [^177^Lu]Lu-PSMA-I&T), ^64^Cu, ^225^Ac, ^211^As, and ^90^Y, has opened the possibility of using them as therapeutic agents [12,13,14,15,16,17,18].

The first attempt at using PSMA as a tumor marker in order to obtain images was done in the form of an ^111^In-labeled monoclonal antibody (mAb)—[^111^In]In-capromab pendetide or ProstaScint^®^—to be used in SPECT tomography. This radiopharmaceutical presented low sensitivity and specificity, mainly because it was aimed at the intracellular domain of PSMA, targeting apoptotic, necrotic, or damaged cells [19,20]. Another mAb was developed to target the extracellular domain of PSMA; however, its applicability is mainly limited because of its long half-life in the circulation system, which has implications for images with high backgrounds or long time periods from injection to imaging [21]. A real advance in image-based PCa diagnosis was reached with the development of the ^68^Ga-labeled PSMA-HBED-CC compound (also named PSMA-11), which presents advantageous characteristics for imaging PCa metastases as a PET radiopharmaceutical [22]. PSMA-11 corresponds to a glutamine–urea–lysine inhibitor, which is conjugated with the highly efficient and gallium-specific *N,N′*-bis-[2-hydroxy-5-(carboxyethyl)benzyl]ethylenediamine-*N,N′*-diacetic acid (HBED-CC) chelator by means of a spacer based on aminohexanoic acid. This urea-based inhibitor presents a high affinity and specificity for PSMA, as well as high and fast internalization to LNCaP cells [8], which is better than that of its corresponding 1,4,7,10-tetraazacyclododecane-1,4,7,10-tetraacetic acid (DOTA)-chelator analogue [23]. Additionally, HBED-CC can be labeled with ^68^Ga in low concentrations and at room temperature. Currently, [^68^Ga]Ga-PSMA-11 is probably the most used radiopharmaceutical for PCa imaging around the world.

In comparison with other radiopharmaceuticals for PCa imaging (e.g., [^11^C]C-Choline, [^18^F]F-Choline, or [^18^F]F-FDG), [^68^Ga]Ga-PSMA-11 has shown improved results. In a study with 37 patients with biochemical relapses of PCa who were evaluated with [^68^Ga]Ga-PSMA-11 and [^18^F]F-fluoromethylcholine PET/Computed Tomography (CT), the former presented a higher detection rate (78 lesions in 32 patients versus 56 lesions in 26 patients; all of these lesions were also detected with [^68^Ga]Ga-PSMA-11), as well as images with higher SUV_max_ and increased contrast (tumor-to-background ratio) in most of the lesions [22]. Based on a literature review and meta-analysis, imaging studies using five different PET radiopharmaceuticals were compared, and, in spite of the limited data, it was verified that tracers targeting PSMA are superior to other radiopharmaceuticals, such as [^18^F]F-FDG, [^11^C]C-Acetate, [^11^C]C- or [^18^F]F-Choline, and anti-1-amino-3-[^18^F]F-fluorocyclobutane-1-carboxylic acid, in detecting extraprostatic lesions [24].

Additional aspects to be considered include accessibility to the radioisotope, production time and procedure, exposure during production, the number of patients that can be attended in the long term, and the effect of the radioisotope on image quality. In this sense, most of these parameters are favored when working with the automatic synthesis module-based production of [^68^Ga]Ga-PSMA-11—like that presented in this work—which provides further advantages for this product [25]. Although there are other possibilities for synthesis, such as direct cyclotron production or manual-based production, automatic synthesis module-based production of [^68^Ga]Ga-PSMA-11 allows very low radiation exposure for technicians, high reproducibility, and compliance with local health regulatory and good manufacturing practice (GMP) standards [26,27]. Furthermore, considering that a ^68^Ge/^68^Ga generator can be eluted from two to three times per day, in-house automatic synthesis module-based production of [^68^Ga]Ga-PSMA-11 provides enough doses for six to eight patients, making it a convenient alternative to direct cyclotron production.

In this sense, the present work aimed to standardize an automatic synthesis module-based radiolabeling protocol for [^68^Ga]Ga-PSMA-11 as a radiopharmaceutical for PET imaging of PCa malignancies that is produced in-house. In vitro and ex vivo assays were performed to determine the characteristics of the radiopharmaceutical in order to validate in-house production while attending to national health regulatory standards.

## 2. Results

### 2.1. Radiolabeling and Radiochemical Purity (RCP) Evaluation

Initially, the radiolabeling protocol was manually evaluated (*n* = 4) previous to the installation of a Modular-Lab PharmTracer synthesis module at the hospital’s radiopharmacy. In this phase, 10 µg/10.56 nmol or 15 µg/15.84 nmol of PSMA-11 were diluted in 1.0 mL of 0.1 M NaOAc buffer with a pH of 4.5. Depending on the activity eluted from the ^68^Ge/^68^Ga generator, 1090 ± 35 MBq of labeled product was obtained, with a labeling yield of 73.1 ± 2.2%, a pH of 4.5, and a radiochemical purity (RCP) of 93.1 ± 2.7%, determined with ascending chromatography, using instant thin layer chromatographic (iTLC) glass microfiber impregnated with silica gel (SG)—iTLC-SG—and 0.1 M NH_4_OAc solution/MeOH (1:1) as eluent.

After appropriate training on using the automatic synthesis module and validation of the quality control methods, routine production was initiated under GMP standards using 20 µg/21.12 nmol of PSMA-11. Radiolabeling yields of >85% were obtained. Data corresponding to the routine production during 2019 (*n* = 30) are illustrated in Table 1.

**Figure 1:**
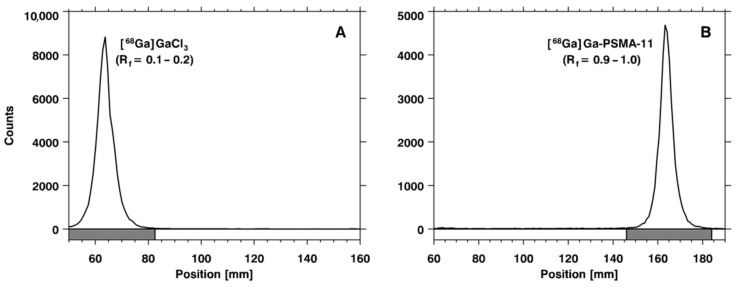
Chromatograms obtained using instant thin layer chromatographic (iTLC) glass microfiber impregnated with silica gel (SG)—iTLC-SG—and 0.1 M NH4OAc solution/MeOH (1:1) as an eluent: (**A**) [68Ga]GaCl3 and (**B**) [68Ga]Ga-PSMA-11. Rf: retention factor.

**Figure 2:**
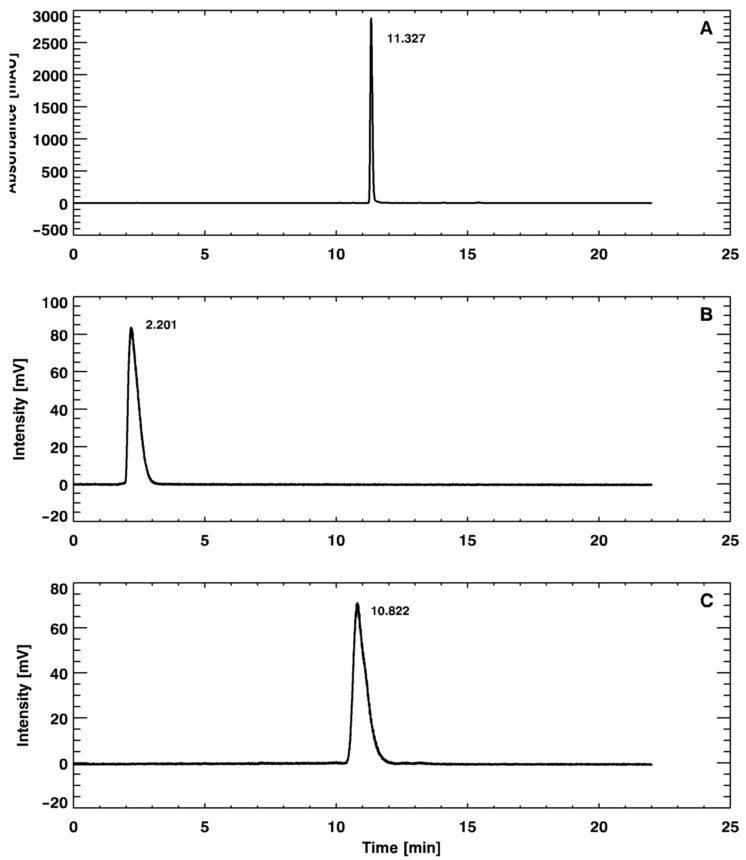
Reversed-phase high-performance liquid chromatography (RP-HPLC) chromatograms of the (**A**) unlabeled precursor Glu-NH-CO-NH-Lys(Ahx)-HBED-CC (PSMA-11), (**B**) [^68^Ga]GaCl_3_, and (**C**) [^68^Ga]Ga-PSMA-11.

In the ascending chromatographic system, free gallium chloride ([^68^Ga]GaCl_3_) remains in the strip origin (retention factor (R_f_) = 0.1–0.2) and [^68^Ga]Ga-PSMA-11 migrates with the solvent front (R_f_ = 0.9–1.0), providing an adequate chromatographic resolution, as illustrated in Figure 1. By using the Sep-Pak C_18_ method, it was possible to quantify the amount of insoluble gallium species or gallium colloids ([^68^Ga]Ga(OH)_x_) retained in the cartridge. Once the Sep-Pak C_18_ method separated both radiochemical impurities, [^68^Ga]GaCl_3_ (collected in the waste vial) and [^68^Ga]Ga(OH)_x_ (retained in the cartridge), the RCP was slightly smaller (~95%) than the value obtained with the iTLC method (~97%), which separated only [^68^Ga]GaCl_3_. The difference between both methods corresponds to the [^68^Ga]Ga(OH)_x_ retained in the Sep-Pak C_18_ cartridge. Both the iTLC-SG and Sep-Pak C_18_ methods were introduced into the routine to assess RCP, considering that their results were compatible with data provided by a reversed-phase high-performance liquid chromatography (RP-HPLC) analysis (Figure 2), which was used to validate the iTLC-SG and Sep-Pak C_18_ assays. Additionally, it is worth mentioning that the iTLC-SG and Sep-Pak C_18_ methods are faster, have lower costs, and are easier to perform in a hospital’s radiopharmacy. Furthermore, all of the batches produced with this standardized automatic synthesis module-based method showed acceptable pH values and were sterile and pyrogen-free.

Operating with two technicians and under the supervision of an experienced radiopharmacist, the synthesis was completed within 15 min. An additional 10 min were required for quality control tests (iTLC-SG, Sep-Pak C_18_, pH, and pyrogen). Overall, the final labeled product was ready to be used in roughly 25 min. Given the properties of the ^68^Ge/^68^Ga generator and according to the number of patients, it is possible to have from two to three productions per day, allowing for six to eight patients to be injected with a dose of 130–222 MBq (3.5–6.0 mCi).

### 2.2. Evaluation of the Stability of the Radiolabeled Compound

The stability of [^68^Ga]Ga-PSMA-11 in saline solution at room temperature was evaluated for up to 4 h by measuring the RCP of the samples using RP-HPLC analysis. The results are presented in Table 2, demonstrating appropriate stability (RCP > 98%) all along this time period. The stability of [^68^Ga]Ga-PSMA-11 in serum at 37 °C was also evaluated for up to 1 h through RP-HPLC analysis. In this case, the RCP values obtained at 0.5 and 1 h were >97% and >95%, respectively (*n* = 3).

### 2.3. In Vitro Characterization

The partition coefficient (P) of [^68^Ga]Ga-PSMA-11 was determined and Log P was calculated as –3.80 ± 0.15 (*n* = 14), confirming the hydrophilic characteristics of the radiopharmaceutical. Beyond that, the serum protein binding (SPB) of [^68^Ga]Ga-PSMA-11 was also determined, and low values were obtained (16.13 ± 1.19% and 16.96 ± 0.44% for mouse and rat, respectively). An unpaired Student *t*-test showed no significant difference (*t* = 1.31, *df* = 7, *p* = 0.2316) between the results for mouse serum (*n* = 5) and rat serum (*n* = 4).

**Figure 3:**
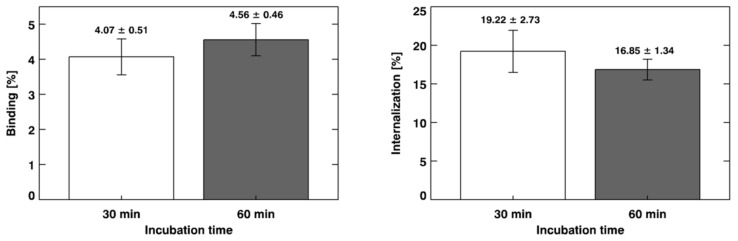
In vitro binding and internalization of [^68^Ga]Ga-PSMA-11 to lymph node carcinoma of the prostate (LNCaP) cells. The results are expressed as “mean ± SD” (*n* = 5).

The in vitro binding and internalization percentages of [^68^Ga]Ga-PSMA-11 to LNCaP cells are summarized in Figure 3. An unpaired Student *t*-test showed no significant difference (binding: *t* = 1.60; *df* = 8; *p* = 0.1493; internalization: *t* = 1.74; *df* = 8; *p* = 0.1196) between the evaluated incubation time intervals (*n* = 5).

### 2.4. Ex Vivo Biodistribution

**Figure 4:**
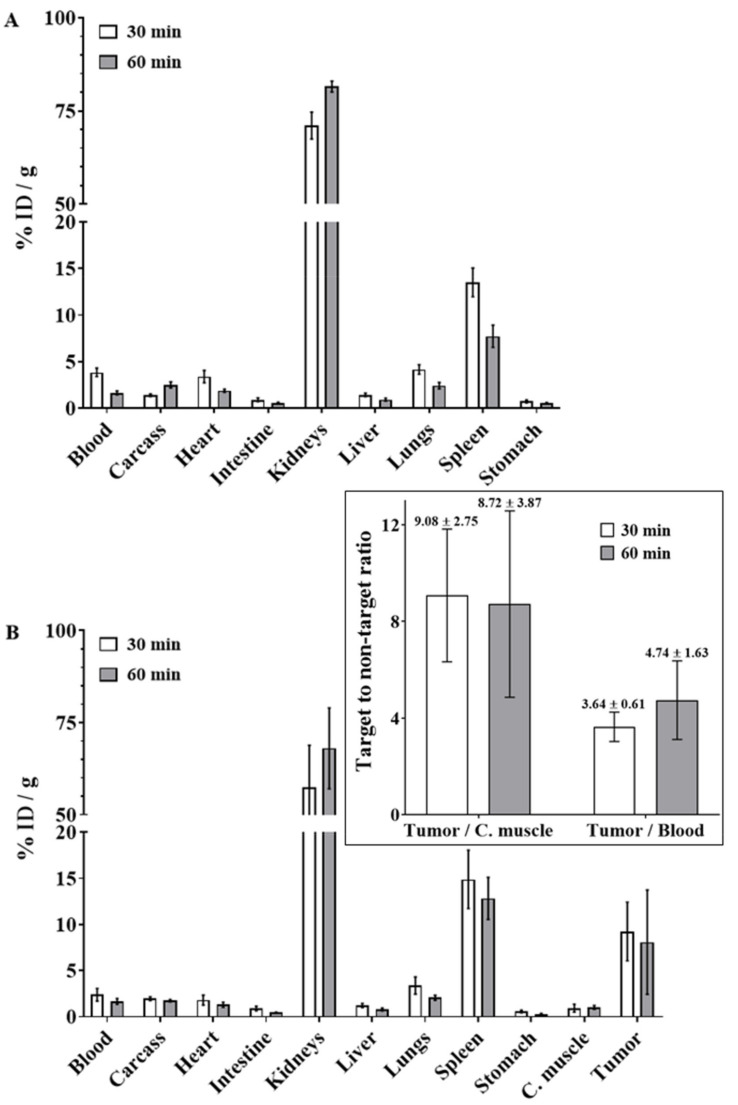
Ex vivo biodistribution profile obtained at 30 and 60 min after intravenous injection of [^68^Ga]Ga-PSMA-11 into (**A**) healthy and (**B**) LNCaP-tumor-bearing mice. Insert: Target-to-non-target ratios calculated with ex vivo biodistribution data obtained in LNCaP-tumor-bearing mice. The values are expressed as “mean ± SD” (n = 5). C. muscle: contralateral muscle.

The ex vivo biodistribution of [^68^Ga]Ga-PSMA-11 was assessed after injection into healthy and LNCaP-tumor-bearing mice (Figure 4). The data showed that the radiopharmaceutical presented rapid blood clearance, followed by high uptake by the kidneys. Beyond that, high [^68^Ga]Ga-PSMA-11 uptake by the spleen was observed. Ex vivo biodistribution data that were obtained in xenografted prostate-tumor-bearing mice showed that [^68^Ga]Ga-PSMA-11 uptake by the tumor was >8% at 30 and 60 min after the radiopharmaceutical administration. No significant difference was observed between the time intervals (*t* = 0.40; *df* = 8; *p* = 0.6989) for tumor uptake (*n* = 5). Target-to-non-target ratios were calculated, and the values were >8.5 and >3.5 for the tumor-to-contralateral-muscle ratio and tumor-to-blood ratio, respectively. In addition, in this case, no significant difference was observed between the evaluated time intervals (tumor-to-contralateral-muscle ratio: *t* = 0.13; *df* = 4; *p* = 0.9026; tumor-to-blood ratio: *t* = 1.10; *df* = 5; *p* = 0.3226) of biodistribution (*n* = 5).

## 3. Discussion

**Figure 5:**
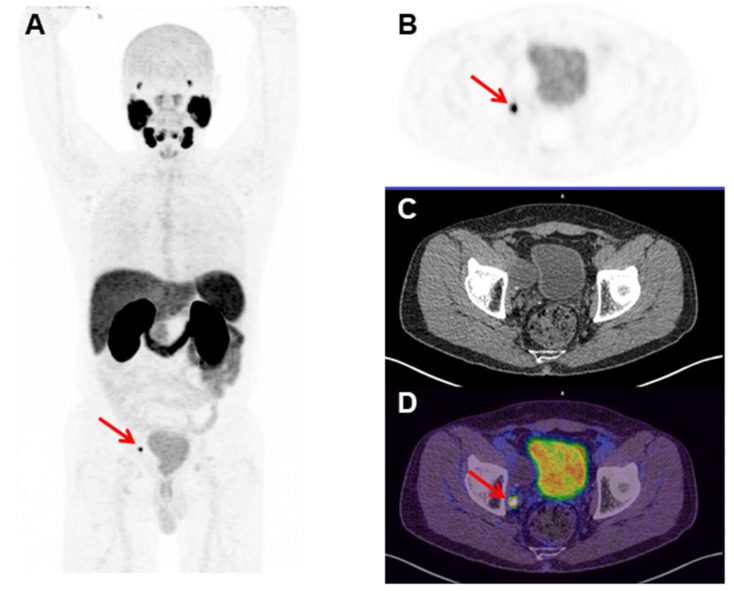
[^68^Ga]Ga-PSMA-11 Positron Emission Tomography (PET) images of a 68 year old man with a Gleason score of 8 (4 + 4), prostate cancer (PCa), and a serum prostate-specific antigen (PSA) of 0.15 ng/mL, who was treated with initial radical prostatectomy four months before the present exam. (A) The maximum intensity projection (MIP), (B) axial PET, (C) Computed Tomography (CT), and (D) fused PET/CT images of the pelvis show a small (6 mm) [^68^Ga]Ga-PSMA-11 PET-positive right obturator lymph node (red arrows) (Nuclear Medicine Department, Hospital Israelita Albert Einstein, Sao Paulo, Brazil).

^68^Ga-labeled molecules must present high RCP values (>90%) to be applicable in a clinical setup [28,29]. The radiolabeling procedure with ^68^Ga can yield free [^68^Ga]GaCl_3_ and [^68^Ga]Ga(OH)_x_, the main radiochemical impurities, which may interfere with imaging interpretation. In this sense, ascending chromatography (using iTLC-SG and 0.1 M NH_4_OAc solution/MeOH (1:1) as an eluent), solid-phase extraction (using a Sep-Pack C_18_cartridge), and an RP-HPLC analysis were performed in order to evaluate the RCP of [^68^Ga]Ga-PSMA-11. The results obtained by the three analytical methods were >95%. A similar RCP for [^68^Ga]Ga-PSMA-11 was previously described [30,31]. Therefore, the data obtained in the present work showed that [^68^Ga]Ga-PSMA-11 was obtained with high RCP with this standardized automatic synthesis module-based method. It is worth mentioning that the automatic synthesis module-based method has many advantages compared to manual-based methods once the automated module is placed inside a hot cell and under laminar flow, allowing high reproducibility, low radiation exposure, and compliance to GMP standards. [^68^Ga]Ga-PSMA-11 was stable in saline solution for up to 4 h (RCP > 98%). These data are in agreement with those that were previously described, which showed in vitro stability of [^68^Ga]Ga-PSMA-11 for up to 3 h [31]. Moreover, [^68^Ga]Ga-PSMA-11 was stable in serum for up to 1 h (RCP > 95%). Thus, the standardized automatic synthesis module-based method is able to produce [^68^Ga]Ga-PSMA-11 with high RCP and suitable in vitro stability. The automated Modular-Lab PharmTracer synthesis module was effectively applied in the routine for [^68^Ga]Ga-PSMA-11 production and is a useful tool for the clinical development of other ^68^Ga-labeled compounds. High-quality [^68^Ga]Ga-PSMA-11 was obtained over four years in more than 1300 syntheses for clinical applications in more than 3000 patients, as exemplified in Figure 5. No adverse reactions were found in this population. Furthermore, clinical applications of [^68^Ga]Ga-PSMA-11 produced by this standardized automatic synthesis module-based method were reported, such as a rare finding of testicular metastasis from PCa [32], the description of an irreversible two-tissue compartment model for the kinetics of this radiopharmaceutical in humans [33], and its high performance for the detection of recurrent PCa after prostatectomy [34].

The P of [^68^Ga]Ga-PSMA-11 was determined by the ratio between *n*-octanol and water. Then, Log P was calculated, and its negative value confirmed the hydrophilic characteristics of [^68^Ga]Ga-PSMA-11 [35]. Furthermore, low SPB values were observed, which were in agreement with the obtained Log *p* value [36]. These results are in accordance with previously published data for the same or similar molecules [37,38,39].

The binding of [^68^Ga]Ga-PSMA-11 to LNCaP cells was evaluated; it represents the radiopharmaceutical molecules that bound to LNCaP cells, including the fraction of molecules that were internalized. This issue can be explained by PSMA expression on the surface of LNCaP cell membranes [9]. Internalization represents the fraction of [^68^Ga]Ga-PSMA-11 that was internalized into LNCaP cells relative to the radiopharmaceutical bound to PSMA expressed on the cell surfaces [40]. The data obtained in the present work are in accordance with those obtained by Ferro-Florez and co-workers for [^99m^Tc]Tc-EDDA/HYNIC-iPSMA binding and internalization in LNCaP cells [41].

The ex vivo biodistribution profile of [^68^Ga]Ga-PSMA-11, which was evaluated in healthy and LNCaP-tumor-bearing mice, showed rapid blood clearance and elimination by the urinary tract, which is consistent with the hydrophilic nature of the molecule (Log *p* < 0) and its low SPB, as previously described. It is important to comment that the substantial [^68^Ga]Ga-PSMA-11 uptake by the spleen observed in our data has also been previously reported in animals [42] as well as in humans [43], although it is not a specific target. The high [^68^Ga]Ga-PSMA-11 uptake by the tumor and, consequently, high tumor-to-contralateral-muscle ratio are explained by the presence of PSMA on the surfaces of LNCaP cells [9]. Therefore, the radiolabeled ligand bound to this antigen and was internalized into the tumor cells according to in vitro assays. On the other hand, the high tumor-to-blood ratio is due to the low SPB of [^68^Ga]Ga-PSMA-11 and its rapid blood clearance and elimination by the urinary tract, as previously discussed. Thus, these results corroborate the in vivo specificity of [^68^Ga]Ga-PSMA-11 for prostate tumor tissue and indicate that this radiopharmaceutical is appropriate for PET images in adequate quality for PCa diagnosis. Considering all of these in vitro and ex vivo data, the standardized automatic synthesis module-based in-house production of [^68^Ga]Ga-PSMA-11 was validated according to national health regulatory standards.

## 4. Materials and Methods

### 4.1. [^68^Ga]Ga-PSMA-11 Radiolabeling Procedure

The synthesis of [^68^Ga]Ga-PSMA-11 was initially carried out manually to determine the optimal synthesis and quality control parameters. Later on, the synthesis protocol was implemented in an automated Modular-Lab PharmTracer synthesis module (Eckert and Ziegler—Berlin, Germany) for routine production. The precursor Glu-NH-CO-NH-Lys(Ahx)-HBED-CC (PSMA-11) and the synthesis cassettes were acquired from ABX Advanced Biochemical Compounds (Radeberg, Germany). The IGG 100 ^68^Ge/^68^Ga generator was obtained from Eckert and Ziegler (Berlin, Germany), which has a guarantee that the ^68^Ge breakthrough is <0.001% over time. Solvents and other reagents were acquired from Merck (Darmstadt, Germany) or Sigma-Aldrich Sweden (Stockholm, Sweden) in high chemical degrees and metal-free media.

[^68^Ga]GaCl_3_ was eluted with 6 mL of 0.1 M HCl solution and purified in a cationic filter. The purified ^68^Ga retained in the filter was then eluted with 0.5 mL of 5 M NaCl solution/5.5 N HCl solution (97:3) into a reaction vial containing 10 to 20 μg of PSMA-11 diluted in 1.0 mL of 0.1 M NaOAc buffer with a pH of 4.5 and heated at 85 °C for 3–5 min. After this reaction time, the [^68^Ga]Ga-PSMA-11 was purified using a Sep-Pak C_18_ cartridge (Waters-Milford, MA, USA) and preconditioned with 5 mL of EtOH/H_2_0 (1:1) solution and 5 mL of 0.9% NaCl solution. The final product was eluted from the Sep-Pak C_18_ cartridge with 0.4 mL of EtOH/H_2_0 (1:1) solution and diluted with 6 mL of 0.9% NaCl solution through a 0.22 μm Cathivex-GV filter (Millipore-Burlington, MA, USA). The labeling yield was determined at the end of the automated synthesis as the ratio between the activity of the final product (purified [^68^Ga]Ga-PSMA-11) vial and the total activity in the module; the latter was obtained as the sum of the activities in the different parts or components of the module (final product vial + empty reaction vial + waste vial + cationic filter + Sep-Pak C_18_ cartridge + 0.22 µm filter). This calculation was made without correcting the activity decay. The activities were measured in a CRC 25 PET dose calibrator (Capintec—New Jersey, NJ, USA). For the integrity filter test, a bubble-point method was performed at the end of the synthesis. The pyrogen was evaluated in all batches with an Endosafe^®^-PTS (Charles River Laboratories—Wilmington, MA, USA) at a dilution factor of 1:50. In order to attest sterility, the microbiological assays were carried out by the hospital’s clinical analysis laboratory.

All equipment used in this work for measurements and quality control assessments was previously calibrated, and preventive maintenance was carried out periodically.

### 4.2. Radiochemical Purity (RCP) Evaluation

The RCP of [^68^Ga]Ga-PSMA-11 was evaluated by ascending chromatography, solid-phase extraction (SPE), and RP-HPLC.

#### 4.2.1. Ascending Chromatography and Solid-Phase Extraction (SPE)

Ascending chromatography was performed with iTLC-SG obtained from Agilent Technologies (Saint Clair, CA, USA), and 0.1 M NH_4_OAc solution/MeOH (1:1) was used as an eluent. After chromatographic development, the radioactivity distribution on the strip was determined with an AR-2000 radio-thin layer chromatography (TLC) imaging scanner (Eckert and Ziegler—Berlin, Germany). The retention factors (R_f_) of [^68^Ga]GaCl_3_ and [^68^Ga]Ga-PSMA-11, as well as the percentage of activity around the corresponding peaks, were determined.

SPE was performed using Sep-Pak C_18_ cartridges (Waters—Milford, MA, USA), preconditioned with 5 mL of EtOH/H_2_0 (1:1) solution and 5 mL of 0.9% NaCl solution. The [^68^Ga]Ga-PSMA-11 was eluted from the Sep-Pak C_18_ cartridge with 0.4 mL of EtOH/H_2_0 (1:1) solution. The activities of the final product ([^68^Ga]Ga-PSMA-11) vial, waste vial (containing [^68^Ga]GaCl_3_), and Sep-Pak C_18_ cartridge (containing the retained [^68^Ga]Ga(OH)_x_) were determined with a CRC 25 PET dose calibrator (Capintec—New Jersey, NJ, USA), and the RCP was determined as follows (Equation (1)):

(1) $$\mathrm{RCP}=\frac{\mathrm{Final}\;\mathrm{product}\;\mathrm{vial}\;\mathrm{activity}\;[\mathrm{Bq}]}{{\mathrm{Total}\;\mathrm{activity}\;(\mathrm{final}\;\mathrm{product}\;\mathrm{vial}\;+\mathrm{waste}\;\mathrm{vial}\;+\;\mathrm{Sep}-\mathrm{Pak}\;C}_{18}\;\mathrm{cartridge})\;[\mathrm{Bq}]}\times 100\%$$

#### 4.2.2. Reversed-Phase High-Performance Liquid Chromatography (RP-HPLC)

RP-HPLC analyses were performed on 1290 Infinity II Ultra High-Performance Liquid Chromatography (UHPLC) equipment (Agilent Technologies—Saint Clair, CA, USA), coupled with a radioactivity detector (Eckert and Ziegler—Berlin, Germany) and equipped with the Open Lab ECM data-processing software (Agilent Technologies—Saint Clair, CA, USA). An analytical column Phenomenex Kinetex^®^ C_18_ (150 mm × 4.6 mm; 5 µm), which was maintained at 25 °C, was used. Mobile phase A was 0.1% trifluoroacetic acid (TFA) in water and mobile phase B was 0.1% TFA in acetonitrile (ACN). The gradients of mobile phase B were: 5–10% (0–2 min), 10–30% (2–16 min), and 30–5% (16–22 min). The flow rate in the system was 1.0 mL⋅min^−1^, and the signals were obtained with a UV detector (λ = 284 nm) and radioactivity detector. The retention times (R_t_) of unlabeled precursor PSMA-11, [^68^Ga]GaCl_3_, and [^68^Ga]Ga-PSMA-11, as well as the percentages of activity of the corresponding peaks, were determined.

### 4.3. Evaluation of the Stability of the Radiolabeled Compound

The stability of [^68^Ga]Ga-PSMA-11 was evaluated in saline solution and in serum. In saline solution (room temperature), this evaluation was performed 1, 2, 3, and 4 h after the radiolabeling procedure. The stability of [^68^Ga]Ga-PSMA-11 in serum—incubated at 37 °C under slight agitation—was evaluated at 0.5 and 1 h post-incubation. Briefly, an aliquot of [^68^Ga]Ga-PSMA-11 (50 µL/~1 MBq) was incubated at 37 °C for 0.5 and 1 h with 450 µL of serum under slight agitation. For each time analyzed, an aliquot was precipitated with cold ACN (1:1), and the sample was centrifuged (825 *g*, 5 min) with Minispin^®^ plus (EppendorfAG—Hamburg, Germany). The supernatant was filtered with a 0.22 µm Millex-GV syringe filter (Merck—Burlington, MA, USA). In both cases—saline solution and serum—aliquots of [^68^Ga]Ga-PSMA-11 were analyzed with RP-HPLC as previously described.

### 4.4. Partition Coefficient (P) Determination

The P of [^68^Ga]Ga-PSMA-11 was determined using the shake flask method, as previously described [44,45]. Briefly, an aliquot of [^68^Ga]Ga-PSMA-11 (50 µL/~1 MBq) was added to a mixture of *n*-octanol/water (1:1) (*n* = 14). The mixture was vigorously agitated (~15 s) and left standing until complete separation of the solvents. Then, aliquots of 100 µL of each phase were collected, and their radioactivities were measured in a Wizard^2^™ 3” 2480 automatic gamma counter (PerkinElmer—Norwalk, CT, USA). Lipophilicity, which is given by the Log P, was calculated as follows (Equation (2)):

(2) $$\log P=\log\left[\frac{\mathrm{cpm}\;(\mathrm{organic}\;\mathrm{phase})}{\mathrm{cpm}\;(\mathrm{aqueous}\;\mathrm{phase})}\right]$$

### 4.5. Serum Protein Binding (SPB) Evaluation

The SPB of [^68^Ga]Ga-PSMA-11 was evaluated as described elsewhere [46]. Briefly, an aliquot of [^68^Ga]Ga-PSMA-11 (50 µL/~1 MBq) was incubated at 37 °C for 60 min with 450 µL of a pool of either mouse serum (*n* = 5) or rat serum (*n* = 4) under slight agitation. After the incubation period, serum proteins were precipitated with cold ACN (1:1), and the samples were centrifuged (825 *g*, 5 min) with a Minispin^®^ plus (EppendorfAG—Hamburg, Germany). The radioactivities of the pellets and supernatants were measured in a Wizard^2^™ 3” 2480 automatic gamma counter (PerkinElmer—Norwalk, CT, USA). The SPB was calculated as follows (Equation (3)):

(3) $$\mathrm{SPB}=\frac{\mathrm{cpm}\;(\mathrm{pellet})}{\mathrm{cpm}\;(\mathrm{pellet}+\mathrm{supernatant})}\times 100\%$$

### 4.6. LNCaP Cell Culture

The LNCaP human cell line was obtained from the *Banco de Células do Rio de Janeiro* (code 0149) (Rio de Janeiro, RJ, Brazil). LNCaP cells (passage 3–6) were grown in RPMI-1640 medium (Sigma-Aldrich—Darmstadt, Germany) and supplemented with 10% (v/v) fetal bovine serum (Sigma-Aldrich—Darmstadt, Germany) and 1% (*v/v*) penicillin and streptomycin antibiotic solution (Sigma-Aldrich—Darmstadt, Germany). The cells were kept in humidified air containing 5% CO_2_ at 37 °C. The cells were grown to confluence and then harvested via trypsinization. After centrifugation (1200 rpm, 5 min), the cells were resuspended in RPMI-1640 medium for in vitro binding and internalization assays or in a 1:1 mixture of Matrigel and RPMI-1640 medium for development of the prostate tumor animal model.

### 4.7. In Vitro Binding to and Internalization of [^68^Ga]Ga-PSMA-11 into LNCaP Cells

Binding to and internalization of [^68^Ga]Ga-PSMA-11 into LNCaP cells were assessed in vitro as previously described [40]. Briefly, an aliquot containing 2 × 10^6^ LNCaP cells in RPMI-1640 medium (450 µL) was added to a vial and incubated with [^68^Ga]Ga-PSMA-11 (50 µL/~1 MBq) at 37 °C and under slight agitation for 30 and 60 min (*n* = 5 for each time interval). Then, the vials were centrifuged (825 *g*, 5 min) with Minispin^®^ plus (EppendorfAG—Hamburg, Germany). The cell pellets and supernatants were separated, and their radioactivities were measured in a Wizard^2^™ 3” 2480 automatic gamma counter (PerkinElmer—Norwalk, CT, USA). Binding to LNCaP cells was calculated as follows (Equation (4)):

(4) $$\mathrm{Binding}=\frac{\mathrm{cpm}\;(\mathrm{pellet})}{\mathrm{cpm}\;(\mathrm{pellet}+\mathrm{supernatant})}\times 100\%$$

After that, pellets were resuspended in 0.5 mL of acid wash buffer (0.2 M acetic acid in 0.5 M NaCl solution, pH 2.8) and kept at room temperature for 5 min to remove cell-surface-bound [^68^Ga]Ga-PSMA-11. Then, the vials were centrifuged (825 *g*, 5 min) with Minispin^®^ plus (EppendorfAG—Hamburg, Germany). The cell pellets and supernatants were separated and their radioactivities were measured in a Wizard^2^™ 3” 2480 automatic gamma counter (PerkinElmer—Norwalk, CT, USA). Internalization into LNCaP cells was calculated as follows (Equation (5)):

(5) $$\mathrm{Internalization}=\frac{\mathrm{cpm}\;(\mathrm{pellet})}{\mathrm{cpm}\;(\mathrm{pellet}+\mathrm{supernatant})}\times 100\%$$

### 4.8. LNCaP Prostate Tumor Animal Model

Male BALB/c nude mice (~20 g/~60 days) were supplied by the vivarium of the *Centro de Experimentação e Treinamento em Cirurgia* of the *Hospital Israelita Albert Einstein* (Sao Paulo, SP, Brazil). The animals were maintained under specific pathogen-free conditions with free access to food and water. The housing suite was temperature (22 ± 2 °C) and humidity (50 ± 10%) controlled with filtered air and a regulated light–dark cycle of 12:12 h, with lights turning on at 07:00 am. All procedures involving mice were conducted in agreement with the Brazilian Guidelines on Care and Use of Animals for Scientific Research or Teaching Activities (DBCA) and were approved by the Ethics Committee on Animal Use of the *Hospital Israelita Albert Einstein*, protocol codes 2843/16 and 3241/17.

In order to produce the tumor model, an aliquot (200 µL) containing 1 × 10^7^ LNCaP cells in a 1:1 mixture of Matrigel and RPMI-1640 medium was injected subcutaneously into the right upper flank of the BALB/c nude mice, as previously described [47], with some modifications. Tumors were allowed to grow in vivo for a maximum period of 40 days after inoculation or until reaching a diameter of 10 mm. LNCaP-tumor-bearing animals were then used for ex vivo biodistribution studies of [^68^Ga]Ga-PSMA-11. Healthy (non-xenografted) male BALB/c nude mice were used as control animals.

### 4.9. [^68^Ga]Ga-PSMA-11 Ex Vivo Biodistribution Studies

LNCaP-tumor-bearing (*n* = 10) and healthy (*n* = 10) male BALB/c nude mice were randomly distributed into two groups for the ex vivo biodistribution study of [^68^Ga]Ga-PSMA-11. Mice were anesthetized with a ketamine–xilazine combination (100:10 mg/Kg) and intravenously injected with an aliquot of ~11.1 MBq of the radiopharmaceutical. At 30 or 60 min after injection (*n* = 5 per time and per group), mice were euthanized. Organs and tissues of interest, such as the blood, carcass, contralateral muscle, heart, intestines, kidneys, liver, lungs, spleen, stomach, and tumor, were dissected and dried on a filter paper. Then, each organ or tissue was weighed, and its associated radioactivity was measured in a Wizard^2^™ 3” 2480 automatic gamma counter (PerkinElmer—Norwalk, CT, USA). A standard dose containing the same amount of radioactivity injected into the mice and defined as 100% was counted simultaneously in a separated tube to correct for the physical decay of ^68^Ga. The data were expressed as the percentage of injected dose per gram of tissue (% ID/g).

### 4.10. Statistical Analysis

Quantitative data were expressed as “mean ± standard deviation (SD)”. Pairs of means were compared using a Student *t*-test. The statistical significance threshold for mean differences was set at 0.05. The data were analyzed using the GraphPad Prism v8.0.2 software (GraphPad Software Inc.—La Joya, CA, USA).

## 5. Conclusions

The production of [^68^Ga]Ga-PSMA-11 in an automatic synthesis module was standardized, resulting in a sterile and endotoxin-free final product with high RCP and stability. All of the synthesis and quality control methods take around 25 min, making them highly reproducible under GMP standards for in-house production in a hospital radiopharmacy for routine PET imaging of PCa according to national health regulatory standards. This automated method allows multiple patient applications using one generator elution. After four years of daily work, more than 3000 patients have benefited from its high accuracy and great clinical value. Quality control methods were also standardized, and RCP evaluation using iTLC-SG and Sep-Pak C_18_ assays was shown to be efficient and fast for routine purposes. Finally, in vitro and ex vivo assays were used to validate the quality of the synthesized product for PET imaging in PCa patients.

## Figures and Tables

**Table 1:** Mean parameters of [^68^Ga]Ga-PSMA-11 in routine production in 2019.

Final Activity [MBq]	842.86 ± 121.00
Labeling Yield [%]	85.35 ± 5.78
RCP|iTLC-SG [%]	97.87 ± 0.91
RCP|Sep-Pak C_18_ [%]	95.59 ± 1.91
RCP|RP-HPLC [%]	99.06 ± 0.10
pH	4.5 ± 0.3
Radioactive Concentration [MBq/mL]	105.35 ± 0.10
Pyrogen	Negative
Filter Integrity|Bubble-point test [bar]	>2.0

Values are expressed as “mean ± SD” (*n* = 30).

**Table 2:** Stability of [^68^Ga]Ga-PSMA-11 in saline solution at room temperature, assessed with RP-HPLC analysis.

Time [h]	RCP [%]
0	99.06 ± 0.10
1	99.02 ± 0.16
2	98.90 ± 0.19
3	98.43 ± 0.15
4	98.10 ± 0.13

Values are expressed as “mean ± SD”: (*n* = 3). RCP: radiochemical purity.

## Data Availability

The data presented in this study are available on request from the corresponding author. The data are not publicly available due to privacy.

## References

[1] Cancer Stat Facts: National Cancer Institute. https://seer.cancer.gov/statfacts/html/prost.html.

[2] Estatísticas de Câncer Instituto Nacional de Câncer. https://www.inca.gov.br/numeros-de-cancer.

[3] Sheikhbahaei S., Afshar-Oromieh A., Eiber M., Solnes L.B., Javadi M.S., Ross A.E., Pienta K.J., Allaf M.E., Haberkorn U., Pomper M.G. (2017). Pearls and pitfalls in clinical interpretation of prostate-specific membrane antigen (PSMA)-targeted PET imaging. Eur. J. Nucl. Med. Mol. Imaging.

[4] Wright G.L., Haley C., Beckett M.L., Schellhammer P.F. (1995). Expression of prostate-specific membrane antigen in normal, benign, and malignant prostate tissues. Urol. Oncol..

[5] Wright G.L., Grob B.M., Haley C., Grossman K., Newhall K., Petrylak D., Troyer J., Konchuba A., Schellhammer P.F., Moriarty R. (1996). Upregulation of prostate-specific membrane antigen after androgen-deprivation therapy. Urology.

[6] Silver D.A., Pellicer I., Fair W.R., Heston W.D., Cordon-Cardo C. (1997). Prostate-specific membrane antigen expression in normal and malignant human tissues. Clin. Cancer Res..

[7] Sweat S.D., Pacelli A., Murphy G.P., Bostwick D.G. (1998). Prostate-specific membrane antigen expression is greatest in prostate adenocarcinoma and lymph node metastases. Urology.

[8] Maurer T., Eiber M., Schwaiger M., Gschwend J.E. (2016). Current use of PSMA-PET in prostate cancer management. Nat. Rev. Urol..

[9] Israeli R.S., Powell C.T., Fair W.R., Heston W.D. (1993). Molecular cloning of a complementary DNA encoding a prostate-specific membrane antigen. Cancer Res..

[10] Chatalic K.L., Heskamp S., Konijnenberg M., Molkenboer-Kuenen J.D., Franssen G.M., Clahsen-van Groningen M.C., Schottelius M., Wester H.-J., Van Weerden W.M., Boerman O.C. (2016). Towards Personalized Treatment of Prostate Cancer: PSMA I&T, a Promising Prostate-Specific Membrane Antigen-Targeted Theranostic Agent. Theranostics.

[11] Lütje S., Heskamp S., Cornelissen A.S., Poeppel T.D., van den Broek S.A., Rosenbaum-Krumme S., Bockisch A., Gotthardt M., Rijpkema M., Boerman O.C. (2015). PSMA Ligands for Radionuclide Imaging and Therapy of Prostate Cancer: Clinical Status. Theranostics.

[12] Weineisen M., Simecek J., Schottelius M., Schwaiger M., Wester H.J. (2014). Synthesis and preclinical evaluation of DOTAGA-conjugated PSMA ligands for functional imaging and endoradiotherapy of prostate cancer. EJNMMI Res..

[13] Weineisen M., Schottelius M., Simecek J., Baum R.P., Yildiz A., Beykan S., Kulkarni H.R., Lassmann M., Klette I., Eiber M. (2015). 68Ga- and 177Lu-Labeled PSMA I&T: Optimization of a PSMA-Targeted Theranostic Concept and First Proof-of-Concept Human Studies. J. Nucl. Med..

[14] Grubmüller B., Baum R.P., Capasso E., Singh A., Ahmadi Y., Knoll P., Floth A., Righi S., Zandieh S., Meleddu C. (2016). Cu-PSMA-617 PET/CT Imaging of Prostate Adenocarcinoma: First In-Human Studies. Cancer Biother. Radiopharm..

[15] Kulkarni H.R., Singh A., Schuchardt C., Niepsch K., Sayeg M., Leshch Y., Wester H.-J., Baum R.P. (2016). PSMA-Based Radioligand Therapy for Metastatic Castration-Resistant Prostate Cancer: The Bad Berka Experience Since 2013. J. Nucl. Med..

[16] Barber T.W., Singh A., Kulkarni H.R., Niepsch K., Billah B., Baum R.P. (2019). Clinical Outcomes of ^177^Lu-PSMA Radioligand Therapy in Earlier and Later Phases of Metastatic Castration-Resistant Prostate Cancer Grouped by Previous Taxane Chemotherapy. J. Nucl. Med..

[17] Emmett L., Crumbaker M., Ho B., Willowson K., Eu P., Ratnayake L., Epstein R., Blanksby A., Horvath L., Guminski A. (2019). Results of a Prospective Phase 2 Pilot Trial of ^177^Lu-PSMA-617 Therapy for Metastatic Castration-Resistant Prostate Cancer Including Imaging Predictors of Treatment Response and Patterns of Progression. Clin. Genitourin. Cancer..

[18] Ruigrok E.A.M., van Weerden W.M., Nonnekens J., de Jong M. (2019). The Future of PSMA-Targeted Radionuclide Therapy: An Overview of Recent Preclinical Research. Pharmaceutics.

[19] Sodee D.B., Ellis R.J., Samuels M.A., Spirnak J.P., Poole W.F., Riester C., Martanovic D.M., Stonecipher R., Bellon E.M. (1998). Prostate cancer and prostate bed SPECT imaging with ProstaScint: Semiquantitative correlation with prostatic biopsy results. Prostate.

[20] Cimadamore A., Cheng M., Santoni M., Lopez-Beltran A., Battelli N., Massari F., Galosi A.B., Scarpelli M., Montironi R. (2018). New Prostate Cancer Targets for Diagnosis, Imaging, and Therapy: Focus on Prostate-Specific Membrane Antigen. Front. Oncol..

[21] Pandit-Taskar N., O’Donoghue J.A., Divgi C.R., Wills E.A., Schwartz L., Gönen M., Smith-Jones P., Bander N.H., Scher H.I., Larson S.M. (2015). Indium 111-labeled J591 anti-PSMA antibody for vascular targeted imaging in progressive solid tumors. EJNMMI Res..

[22] Afshar-Oromieh A., Zechmann C.M., Malcher A., Eder M., Eisenhut M., Linhart H.G., Holland-Letz T., Hadaschik B.A., Giesel F.L., Debus J. (2014). Comparison of PET imaging with a (68)Ga-labelled PSMA ligand and (18)F-choline-based PET/CT for the diagnosis of recurrent prostate cancer. Eur. J. Nucl. Med. Mol. Imaging.

[23] Wester H.J., Schottelius M. (2019). PSMA-Targeted Radiopharmaceuticals for Imaging and Therapy. Semin. Nucl. Med..

[24] Yu C.Y., Desai B., Ji L., Groshen S., Jadvar H. (2014). Comparative performance of PET tracers in biochemical recurrence of prostate cancer: A critical analysis of literature. Am. J. Nucl. Med. Mol. Imaging.

[25] Szydlo M., Pogoda D., Kowalski T., Pocięgiel M., Jadwiński M., d’Amico A. (2018). Synthesis and Quality Control of ^68^Ga-PSMA PET/CT Tracer used in Prostate Cancer Imaging and Comparison with ^18^F-Fluorocholine as a Reference Point. J. Pharm. Sci. Emerg. Drugs.

[26] Velikyan I. (2015). 68Ga-Based radiopharmaceuticals: Production and application relationship. Molecules.

[27] Rodnick M.E., Sollert C., Stark D., Clark M., Katsifis A., Hockley B.G., Parr D.C., Frigell J., Henderson B.D., Abghari-Gerst M. (2020). Cyclotron-based production of 68Ga, [68Ga]GaCl3, and [68Ga]Ga-PSMA-11 from a liquid target. EJNMMI Radiopharm. Chem..

[28] Cardinale J., Martin R., Remde Y., Schäfer M., Hienzsch A., Hübner S., Zerges A.-M., Marx H., Hesse R., Weber K. (2017). Procedures for the GMP-Compliant Production and Quality Control of [^18^F]PSMA-1007: A Next Generation Radiofluorinated Tracer for the Detection of Prostate Cancer. Pharmaceuticals.

[29] Edition EPt (2019). European Directorate for the Quality of Medicines & Healthcare. Gallium (68Ga) PSMA-11 Injection. Monograph Number 2485.

[30] Nanabala R., Anees M.K., Sasikumar A., Joy A., Pillai M.R. (2016). Preparation of [(68)Ga]PSMA-11 for PET-CT imaging using a manual synthesis module and organic matrix based (68)Ge/(68)Ga generator. Nucl. Med. Biol..

[31] Lütje S., Franssen G.M., Herrmann K., Boerman O.C., Rijpkema M., Gotthardt M., Heskamp S. (2019). In Vitro and In Vivo Characterization of an 18F-AlF-Labeled PSMA Ligand for Imaging of PSMA-Expressing Xenografts. J. Nucl. Med..

[32] da Cunha M.L., Rodrigues C.d.O., de Araújo M.P.L., de Freitas Junior C.H., Ferrigno R. (2018). Solitary testicular metastasis from prostate cancer. A case report diagnosed by PET/CT with PSMA. Eur. J. Nucl. Med. Mol. Imaging.

[33] Ringheim A., Campos Neto G.C., Anazodo U., Cui L., da Cunha M.L., Vitor T., Martins K.M., Miranda A.C.C., De Barboza M.F., Fuscaldi L.L. (2020). Kinetic modeling of 68Ga-PSMA-11 and validation of simplified methods for quantification in primary prostate cancer patients. EJNMMI Res..

[34] Afshar-Oromieh A., da Cunha M.L., Wagner J., Haberkorn U., Debus N., Weber W., Eiber M., Holland-Letz T., Rauscher I. (2021). Performance of [68Ga]Ga-PSMA-11 PET/CT in patients with recurrent prostate cancer after prostatectomy—a multi-centre evaluation of 2533 patients. Eur. J. Nucl. Med. Mol. Imaging.

[35] Silverman R.B., Holladay M.W. (2015). The organic Chemistry of Drug Design and Drug Action.

[36] Croom E. (2012). Metabolism of xenobiotics of human environments. Prog. Mol. Biol. Transl. Sci..

[37] Trencsényi G., Dénes N., Nagy G., Kis A., Vida A., Farkas F., Szabó J.P., Kovács T., Berényi E., Garai I. (2017). Comparative preclinical evaluation of ^68^Ga-NODAGA and ^68^Ga-HBED-CC conjugated procainamide in melanoma imaging. J. Pharm. Biomed. Anal..

[38] Xu X., Zhang J., Hu S., He S., Bao X., Ma G., Luo J., Cheng J., Zhang Y. (2017). Tc-labeling and evaluation of a HYNIC modified small-molecular inhibitor of prostate-specific membrane antigen. Nucl. Med. Biol..

[39] Young J.D., Abbate V., Imberti C., Meszaros L.K., Ma M.T., Terry S.Y.A., Hider R.C., Mullen G.E., Blower P.J. (2017). Ga-THP-PSMA: A PET Imaging Agent for Prostate Cancer Offering Rapid, Room-Temperature, 1-Step Kit-Based Radiolabeling. J. Nucl. Med..

[40] Fuscaldi L.L., de Barros A.L.B., Santos C.R.d.P., de Oliveira M.C., Fernandes S.O.A., Cardoso V.N. (2015). Feasibility of the ^99m^Tc-HYNIC-βAla-Bombesin_(7–14)_ for detection of LNCaP prostate tumour in experimental model. J. Radioanal. Nucl. Chem..

[41] Ferro-Flores G., Luna-Gutiérrez M., Ocampo-García B., Santos-Cuevas C., Azorín-Vega E., Jiménez-Mancilla N., Orocio-Rodríguez E., Davanzo J., García-Pérez F.O. (2017). Clinical translation of a PSMA inhibitor for ^99m^Tc-based SPECT. Nucl. Med. Biol..

[42] Kopka K., Benešová M., Bařinka C., Haberkorn U., Babich J. (2017). Glu-Ureido-Based Inhibitors of Prostate-Specific Membrane Antigen: Lessons Learned During the Development of a Novel Class of Low-Molecular-Weight Theranostic Radiotracers. J. Nucl. Med..

[43] Ferreira G., Iravani A., Hofman M.S., Hicks R.J. (2019). Intra-individual comparison of ^68^Ga-PSMA-11 and ^18^F-DCFPyL normal-organ biodistribution. Cancer Imaging.

[44] Waterhouse R.N. (2003). Determination of lipophilicity and its use as a predictor of blood-brain barrier penetration of molecular imaging agents. Mol. Imaging Biol..

[45] Durante A.C.R., Sobral D.V., Miranda A.C.C., de Almeida É.V., Fuscaldi L.L., de Barboza M.R.F.F., Malavolta L. (2019). Comparative Study of Two Oxidizing Agents, Chloramine T and Iodo-Gen. Pharmaceuticals.

[46] Sobral D.V., Fuscaldi L.L., Durante A.C.R., Rangel M.G., Oliveira L.R., Mendonça F.F., Miranda A.C.C., Cabeza J.M., Montor W.R., Cabral F.R. (2020). Radiochemical and biological properties of peptides designed to interact with EGF receptor: Relevance for glioblastoma. Nucl. Med. Biol..

[47] Fuscaldi L.L., de Barros A.L.B., de Paula Santos C.R., de Souza C.M., Cassali G.D., de Oliveira M.C., Fernandes S.O.A., Cardoso V.N. (2014). Evaluation of the optimal LNCaP prostate tumour developmental stage to be assessed by 99mTc-HYNIC-βAla-Bombesin(7–14) in an experimental model. J. Radioanal. Nucl. Chem..

